# Risk factors for dementia among indigenous africans: Data from the recruitment and retention for Alzheimer'S Disease diversity genetic cohorts in the ADSP (READD‐ADSP) study

**DOI:** 10.1002/alz70860_107688

**Published:** 2025-12-23

**Authors:** Rufus O. Akinyemi, Brian W Kunkle, Joshua O Akinyemi, Nicholas A. Kushch, Oladotun V Olalusi, Farid Rajabli, Gabriel O Ogunde, Izri M Martinez, Andrew F Zaman, Fred Stephen Sarfo, Albert Akpalu, Alfred K Njamnshi, Albertino Damasceno, Biniyam A Ayele, Yared Zenebe, Thierry Adoukonou, Jean N Ikanga, Judith Boshe, David Ndetei, Reginald Obiako, Adefolakemi Ogundele, Kolawole Wahab, Godwin Osaigbovo, Paul Nwani, Damas Andrea Mlaki, Kamada Lwere, Paul Olowoyo, Olufisayo Oluyinka Elugbadebo, Mayowa Ogunronbi, Oyedunni Arulogun, Michelle Nichols, Stella‐Maria Paddick, Temitope Hannah Farombi, Njideka U Okubadejo, Richard Walker, Olusegun Baiyewu, Mayowa O Owolabi, Goldie S Byrd, Azizi A Seixas, Maëlenn Guerchet, Sudha Seshadri, Mike Cuccaro, Jeffery M Vance, Christiane Reitz, William S Bush, Jonathan L Haines, Raj N Kalaria, Margaret Pericak‐Vance, Adesola Ogunniyi

**Affiliations:** ^1^ College of Medicine, University of Ibadan, Ibadan, Oyo State, Nigeria; ^2^ Africa Dementia Consortium, Ibadan, Ibadan, Nigeria; ^3^ Institute of Advanced Medical Research and Training, College of Medicine, University of Ibadan, Ibadan, Oyo State, Nigeria; ^4^ John P. Hussman Institute for Human Genomics, Miller School of Medicine, University of Miami, Miami, FL, USA; ^5^ College of Medicine, University of Ibadan, Ibadan, Oyo, Nigeria; ^6^ Neuroscience And Ageing Research Unit, Institute for Advanced Medical Research and Training, College of Medicine, University of Ibadan, Ibadan, Nigeria; ^7^ John P. Hussman Institute for Human Genomics, University of Miami Miller School of Medicine, Miami, FL, USA; ^8^ Institute for Advanced Medical Research and Training, College of Medicine, University of Ibadan, Ibadan, Oyo State, Nigeria; ^9^ Department of Neurology, University College Hospital, Ibadan, Oyo, Nigeria; ^10^ Institute for Human Genomics and the Dr. John T. Macdonald Foundation Department of Human Genetics, University of Miami Miller School of Medicine, Miami, FL, USA; ^11^ Institute for Advanced Medical Research and Training, College of Medicine, University of Ibadan, Ibadan, Oyo, Nigeria; ^12^ University of Miami Miller School of Medicine, Miami, FL, USA; ^13^ Kwame Nkrumah University of Science and Technology, Kumasi, Ghana, Kumasi, Ghana; ^14^ University of Ghana Medical School, Accra, Ghana; ^15^ Brain Research Africa Initiative, Yaounde, Centre, Cameroon, Yaounde, Centre, Cameroon, Cameroon; ^16^ Faculty of Medicine, Eduardo Mondlane University, Maputo, Mozambique; ^17^ College of Health Sciences, Addis Ababa University, Addis Ababa, Ethiopia; ^18^ College of Health Sciences, Addis Ababa University, Addis Ababa, Ethiopia, Addid Ababa, Ethiopia; ^19^ Department of Neurology, University of Parakou, Parakou, Benin, Parakou, Parakou, Benin; ^20^ University of Kinshasa, Kinshasa, Kinshasa, Congo (The Democratic Republic of the); ^21^ Kilimanjaro Christian Medical University College, Moshi, Tanzania, United Republic of; ^22^ Africa Mental Health Research and Training Foundation, Nairobi, Kenya; ^23^ Department of Medicine, Ahmadu Bello University, Zaria, Nigeria., Zaria, Nigeria; ^24^ Psychogeriatric Unit, Neuropsychiatric Hospital, Aro, Abeokuta, Nigeria, Abeokuta, Abeokuta, Nigeria; ^25^ Department of Medicine, University of Ilorin, Ilorin, Nigeria; ^26^ Jos University Teaching Hospital, Jos, Plateau, Nigeria; ^27^ Nnamdi Azikiwe University Teaching Hospital, Anambra State, Nigeria, Nnewi, Anambra, Nigeria; ^28^ Mirembe National Mental Health Hospital, Dodoma, Dodoma, Tanzania, United Republic of; ^29^ Makerere University in Uganda, Kampala, Uganda, Uganda; ^30^ Ekiti State University Teaching Hospital, Ekiti, Ekiti, Nigeria; ^31^ Department of Psychiatry, University of Ibadan, Ibadan, Oyo, Nigeria; ^32^ University of Ibadan, Ibadan, Oyo, Nigeria; ^33^ University of South Carolina, Charleston, SC, USA; ^34^ Translational and Clinical Research Institute, Newcastle University, UK/Gateshead Health NHS Foundation Trust, Gateshead, UK, Gateshead, United Kingdom; ^35^ University College Hospital, Ibadan, Nigeria; ^36^ College of Medicine, University of Lagos, Lagos, Nigeria; ^37^ Translational and Clinical Research Institute, Newcastle University, Newcastle, Newcastle Upon Tyne, United Kingdom; ^38^ Wake Forest University School of Medicine, Winston‐Salem, NC, USA; ^39^ Department of Psychiatry and Behavioral Sciences, University of Miami Miller School of Medicine, Miami, FL, USA; ^40^ Department of Informatics and Health Science Data, University of Miami Miller School of Medicine, Miami, FL, USA; ^41^ Univ. Limoges, UMR_S 1094, Tropical Neuroepidemiology, Institute of Neuroepidemiology and Tropical Neurology, CNRS FR 3503 GEIST, F‐87000, Limoges, France; ^42^ Glenn Biggs Institute for Alzheimer's & Neurodegenerative Diseases, University of Texas Health Science Center, San Antonio, TX, USA; ^43^ Institute for Human Genomics and the Dr. John T. Macdonald Foundation Department of Human Genetics, University of Miami Miller School of Medicine, Miami, Florida, USA, Miami, FL, USA; ^44^ Dr. John T. Macdonald Foundation Department of Human Genetics, University of Miami Miller School of Medicine, Miami, FL, USA; ^45^ The Taub Institute for Research on Alzheimer's Disease and The Aging Brain, Columbia University, New York, NY, USA; ^46^ Cleveland Institute for Computational Biology, Department of Population & Quantitative Health Sciences, School of Medicine, Case Western Reserve University, Cleveland, OH, USA; ^47^ Newcastle University, Newcastle, United Kingdom; ^48^ John P. Hussman Institute for Human Genomics, University of Miami, Miami, FL, USA

## Abstract

**Background:**

In 2024, the Lancet Commission on Dementia published another iteration of risk factors and observed that almost half of the burden of dementia is potentially preventable by tackling 14 modifiable risk factors. We performed a preliminary analysis of the risk factors in the African datasets of the ongoing Recruitment and Retention for Alzheimer's Disease Diversity Genetic Cohorts in the ADSP (READD‐ADSP) study.

**Method:**

The READD‐ADSP is an ongoing case‐control study recruiting and evaluating 13,000 participants consisting of 5,000 Africans, 4,000 Black Americans, and 4,000 Hispanic/Latino individuals to elucidate the complete genetic background of AD for the development of global treatments and preventions. The African Dementia Consortium (AfDC) is coordinating ongoing recruitment in ten sub‐Saharan African countries. Protocols were developed to ensure standardized data collection, phenotype harmonization, and culturally informed diagnostic algorithms. We present results of preliminary analysis exploring 12 of the Lancet Commission's 14 modifiable risk factors (excluding LDL‐c and air pollution). A multivariable logistic regression analysis was used to assess the effect sizes of the dementia risk factors in the study population.

**Result:**

Data consisted of 1469 participants (626 cases and 843 controls; mean age 75.3 =/‐9.5 yrs; 41.5% male). Mean age of the cases was 77.0±9.5years while that of controls was 74.1±9.2years (*p* <0.001). Older age, female sex, hypertension, hearing loss, depression, physical inactivity, low level of education, and social isolation were significantly more prevalent among cases compared to controls. However, self‐reported Visual loss was more prevalent in the control population. Factors independently associated with dementia in this analysis include age (AOR 1.03: 1.02‐1.04), hypertension (AOR=1.35; 1.06‐1.72) alcohol use (AOR=0.34, 0.13‐0.87), depression (AOR=2.60; 2.09‐3.23) and self‐reported visual impairment (AOR=0.62; 0.48‐0.80). Age, sex, hypertension, and alcohol exhibit region‐specific differences in magnitude and direction between East and West Africa, while depression and self‐reported visual impairment show consistent effects. However, potential confounding remains a concern due to cross‐regional variations in some factors.

**Conclusion:**

Our findings highlight the unique contributions of multi‐modal risk factors to dementia burden among Africans. As additional data are collected, further analyses will examine the interactive effects of region, ethnicity and other factors.

